# Combining Functional Units to Design Organic Materials with Dynamic Room-Temperature Phosphorescence under Continuous Ultraviolet Irradiation

**DOI:** 10.3390/molecules29112621

**Published:** 2024-06-02

**Authors:** Meng Liu, Zhiqiang Yang, Zhe Feng, Ningyuan Zhao, Ruihua Bian, Jinpu Wu, Qing Yang, Shuaiqiang Zhao, Haichao Liu, Bing Yang

**Affiliations:** 1 State Key Laboratory of Supramolecular Structure and Materials, College of Chemistry, Jilin University, Changchun 130012, China; 2 College of Chemistry, Jilin University, Changchun 130012, China15391069289@163.com (J.W.); 3 State Key Laboratory of Superhard Materials, College of Physics, Jilin University, Changchun 130012, China

**Keywords:** organic room-temperature phosphorescence, thianthrene, folding-induced spin–orbit coupling enhancement, photo response, functional unit combination strategy

## Abstract

Developing materials with dynamic room-temperature phosphorescence (RTP) properties is crucial for expanding the applications of organic light-emitting materials. In this study, we designed and synthesized two novel RTP molecules by combining functional units, incorporating the folded unit thianthrene into the classic luminescent cores thioxanthone or anthraquinone to construct TASO and TA2O. In this combination, the TA unit contributes to the enhancement of spin–orbit coupling (SOC), while the luminescent core governs the triplet energy level. After the strategic manipulation of SOC using the thianthrene unit, the target molecules exhibited a remarkable enhancement in RTP performance. This strategy led to the successful development of TASO and TA2O molecules with outstanding dynamic RTP properties when exposed to continuous ultraviolet irradiation, a result that can be ascribed to their efficient RTP, improved absorption ability, and oxygen-sensitive RTP properties. Leveraging the oxygen-mediated ultraviolet-radiation-induced RTP enhancement in TASO-doped polymer films, we developed a novel time-resolved detection technique for identifying phase separation in polymers with varying oxygen permeability. This research offers a promising approach for constructing materials with dynamic RTP properties.

## 1. Introduction

In recent years, purely organic room-temperature phosphorescent (RTP) materials have seen widespread application in various areas, including organic light-emitting diodes [1,2], anti-counterfeiting and encryption [3,4], sensing [5,6], biological imaging [7,8], and photodynamic therapy [9]. Their growing popularity can be attributed to their long afterglow, easy preparation, and cost-effectiveness [10,11,12]. However, these RTP materials often face challenges related to low RTP emission efficiency due to their weak spin–orbit coupling (SOC) and the forbidden nature of triplet transitions [13,14]. To enhance the performance of purely organic RTP materials, two key strategies have emerged: promoting the intersystem crossing (ISC) process by enhancing SOC and suppressing non-radiative pathways by restricting molecular motions [15,16,17,18]. Most purely organic RTP materials exhibit good RTP properties under ‘static’ conditions but rarely do so under ‘dynamic’ conditions. Nevertheless, the ‘dynamic’ properties hold potential for expanding the applications of purely organic RTP materials [19,20,21].

Photo-induced dynamically responsive RTP enhancement is an emerging phenomenon with various applications like anti-counterfeiting and encryption [22], optical switching [23], and polymer crack detection [24]. Different methods, including oxygen consumption [25], molecular conformation changes in crystals [26,27], polymer matrix cross-linking [28,29], and molecular aggregation [30,31], can be employed to achieve this effect. Among these, oxygen-mediated RTP enhancement in thin films is particularly notable due to the ease of processing and handling compared to brittle crystals. However, there is a scarcity of examples in this area and a lack of well-defined design principles.

In this study, we implemented a functional unit combination strategy [24,32] for the design of materials with photo-induced RTP enhancement. Specifically, a unit is focused on enhancing SOC to promote the ISC process, while another unit dominates the triplet energy level. For this purpose, the luminescent cores, thioxanthone (TX) and anthraquinone (AQ), were modified by incorporating a folding unit, thianthrene (TA) [33,34,35,36]. While both TX and AQ exhibit very weak RTP in their monomeric state [37], this strategic combination transforms their initially weak RTP into strong RTP in the target compounds. This transformation can be ascribed to the more efficient ISC pathways generated by closely aligning singlet–triplet energy levels and increasing SOC coefficients between the S_1_ and T_n_ (n ≥ 1) states for the target compounds. This molecular design also enables the original luminescent core, TX or AQ, to dominate the triplet energy level through controlling charge-transfer (CT) strength, facilitating control over the RTP emission color. The two newly obtained target compounds exhibit oxygen-sensitive RTP properties and a significant RTP enhancement due to oxygen consumption under continuous ultraviolet (UV) irradiation. By leveraging the photo-responsive RTP enhancement property of polymer films, a novel time-resolved photo-responsive detection method for phase separation was developed.

## 2. Results and Discussions

### 2.1. Photophysical Properties

**Scheme 1:**
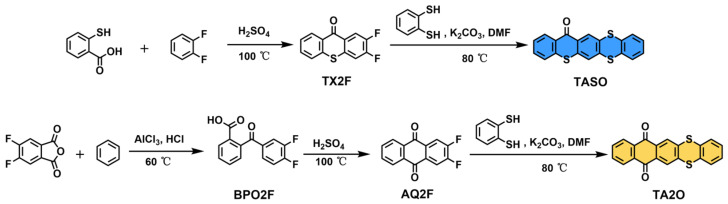
Synthetic routes of TASO and TA2O.

To gain an insight into the availability of functional unit combination strategies, two target compounds, 14H-thiochromeno[2,3-*b*]thianthren-14-one (TASO) and naphtho[2,3-*b*]thianthrene-7,12-dione (TA2O), were synthesized through a ring-formed nucleophilic aromatic substitution reaction [38], as shown in Scheme 1. The synthesis details are provided in the Supplemenatary Materials.

Previous studies conducted by our research group focused on investigating the phosphorescent properties of TX and its derivatives [37]. When TX molecules were doped into a polymethyl methacrylate (PMMA) film at a mass ratio of 1.0%, only fluorescence emission peaking at about 420 nm was observed. To achieve phosphorescent emission, a low-temperature or deoxygenated environment is necessary to suppress non-radiative processes [37]. Similarly, the emission characteristics of PMMA-doped films containing single molecules of AQ were found to be comparable to those of TX, with no RTP observed under ambient conditions (Figure S1).

**Figure 1:**
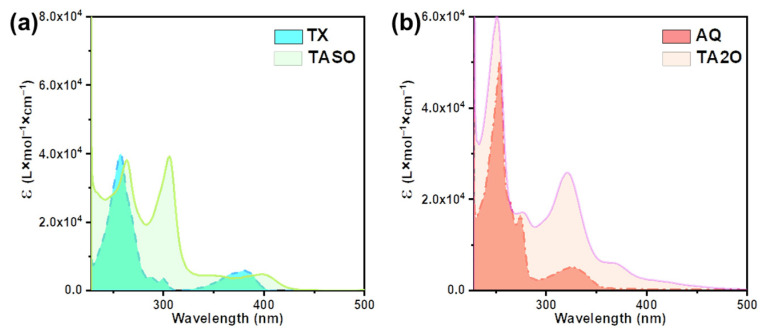
Molar extinction coefficients of (**a**) TX and TASO and (**b**) AQ and TA2O.

To compare the light absorption properties of the target RTP materials, we measured the molar extinction coefficients (*ε*) of the parent cores (TX and AQ) and the target RTP compounds (TASO and TA2O), as illustrated in Figure 1. It was found that the TASO and TA2O compounds exhibit red-shifted absorption band edges compared to their parent phosphorescent cores (TX and AQ), and they also display broader and more intense absorption bands. Obviously, the light from the widely used commercial UV lamp with a wavelength of 365 nm aligns well with the strong absorption near 365 nm of TASO and TA2O, making them highly suitable for various UV light response applications. Additionally, solvatochromism was obtained for these materials (Figure S2). As the solvent polarity increases, the emission peaks of the TASO and TA2O gradually shift towards the long wavelength, indicating the presence of intramolecular CT [39]. TA2O exhibits a larger red shift, signifying a stronger CT effect, which may influence the phosphorescence emission color.

**Figure 2:**
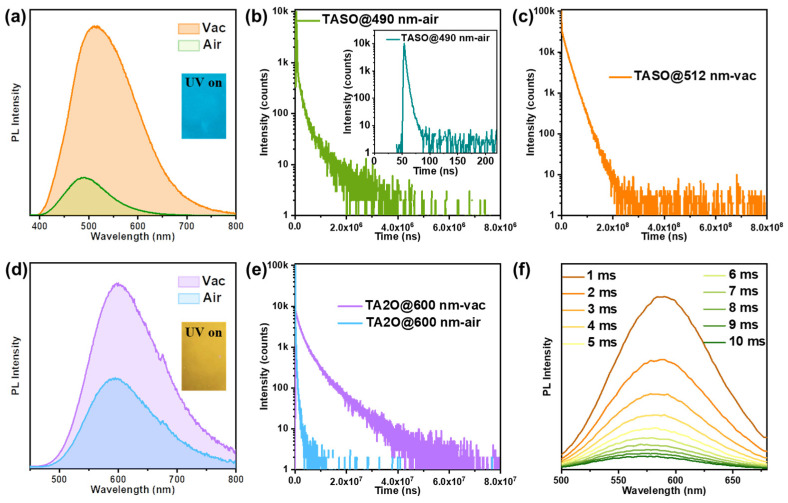
The 1.0 wt.% TASO-doped PMMA film: (**a**) the emission spectra before and after deoxygenation, and the inset is an image of TASO-doped PMMA film under 365 nm UV irradiation in air; (**b**) the time-resolved emission spectrum before deoxygenation; (**c**) the time-resolved emission spectrum after deoxygenation. The 1.0 wt.% TA2O doped PMMA film: (**d**) the emission spectra before and after deoxygenation, and the inset is an image of TA2O-doped PMMA film under 365 nm UV irradiation in air; (**e**) the time-resolved emission spectra before and after deoxygenation; (**f**) the emission spectra with increasing delayed time after deoxygenation.

In order to investigate the RTP properties of TASO and TA2O, we doped them into the PMMA matrix at a mass ratio of 1.0%. Dispersed thin films were prepared on dry and clean quartz sheets using the drop-coating method for further analysis of the subsequent photophysical properties. Through testing the emission spectra, it was observed that the TASO-doped PMMA film exhibited a single emission band both before and after deoxygenation, showing high sensitivity to oxygen (Figure 2a). In air, the TASO-doped thin film displayed lifetimes at both nanosecond and microsecond levels (3.27 ns and 0.49 ms, respectively) at the primary peak position of 490 nm (Figure 2b). Following deoxygenation, the emission spectrum of the TASO-doped film exhibited a slight red shift to 512 nm, increasing from the 490 nm recorded in air, and the lifetime near 512 nm extended to 20.10 ms (Figure 2c). To clarify the luminescent properties of TASO, we conducted temperature-dependent tests of emission and time-resolved emission spectra on the doped film (Figure S3). At low temperatures, the emission spectrum red-shifted from its position at room temperature to 523 nm and was accompanied by a shoulder emission band at a short wavelength. This shoulder band displayed a gradually increasing delayed component upon increasing the temperature, according to the temperature-dependent time-resolved emission spectra, indicating its thermally activated delayed fluorescence (TADF) characteristics [40]. Moreover, the temperature-dependent time-resolved emission spectra monitored at 523 nm show a gradually decreased decay with increasing temperature, demonstrating the phosphorescence characteristic of TASO at 523 nm. These findings align well with the test results for TASO in a diluted solution at low temperature (Figure S4). These experimental results indicate that, in comparison to the parent TX, TASO exhibits TADF properties in air and RTP properties upon deoxygenation conditions. The ratio of the emission peak intensity after deoxygenation to that before deoxygenation (*I*_vac_/*I*_air_) was found to be as high as 5.0, with a significant difference in photoluminescence quantum yield (PLQY) values amounting to 55.49% and 5.72%, respectively. These PLQY values are far higher than those of the parent TX [37].

The TA2O-doped film shows a single peak emission at about 600 nm both before and after deoxygenation, with an extended phosphorescence lifetime observed from 0.39 ms to 6.47 ms after deoxygenation (Figure 2d,e). The temperature-dependent emission and time-resolved emission spectra reveal a decrease in emission intensity and a reduction in the millisecond-level lifetime upon increasing the temperature (Figures S5 and S6), indicative of pure phosphorescence emission from TA2O. Notably, the emission spectra of TA2O remain nearly unchanged at various delay times (Figure 2f), indicating the high stability of the RTP emission. Deoxygenation significantly improved the PLQY from 7.82% to 16.59%. This improved performance was credited to the modification of AQ through the folding unit TA.

### 2.2. Theoretical Calculations

**Figure 3:**
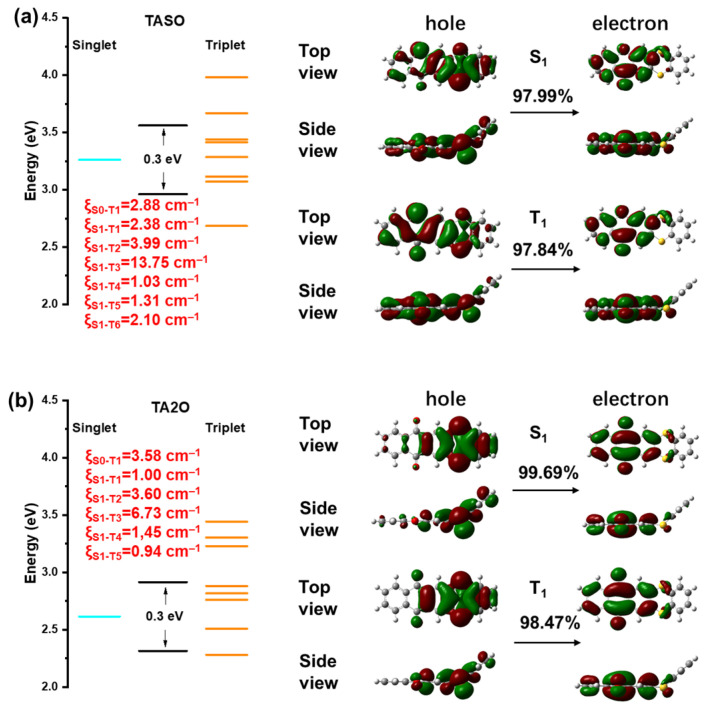
SOC and energy level distribution: (**a**) TASO; (**b**) TA2O.

Following the introduction of TA units, the RTP performance of TASO and TA2O showed a significant enhancement, prompting a deeper investigation into their theoretical mechanisms. Theoretical calculations revealed that, in comparison to the parent TX or AQ, TASO and TA2O possess triplet energy levels more closely aligned with the S_1_ energy level (Figure 3 and Figure S7). Moreover, the overall SOC coefficients significantly improved, indicating a stronger ISC capability in TASO and TA2O. In terms of energy levels, TASO and TA2O display decreasing S_1_ and T_1_ energy levels to varying degrees compared to TX and AQ, with the S_1_ energy level experiencing a more pronounced decrease. This phenomenon can be elucidated through an analysis of their natural transition orbitals (NTOs). Upon the introduction of the TA unit, CT transitions are generated in both TASO and TA2O. Here, the TA unit serves as the donor, while the TX or AQ acts as the acceptor. Notably, TA2O exerts a more pronounced CT effect, indicating that AQ possesses a stronger electron-attracting ability compared to TX. The presence of the CT state allows for adjustment of the triplet energy level, which reinforces the idea that the parent core primarily influences the T_1_ energy level and ultimately dictates the emission color of RTP. The incorporation of the folding unit TA primarily serves to regulate SOC [24], aligning well with our overarching design principles.

### 2.3. Photo-Responsive RTP Enhancement

**Figure 4:**
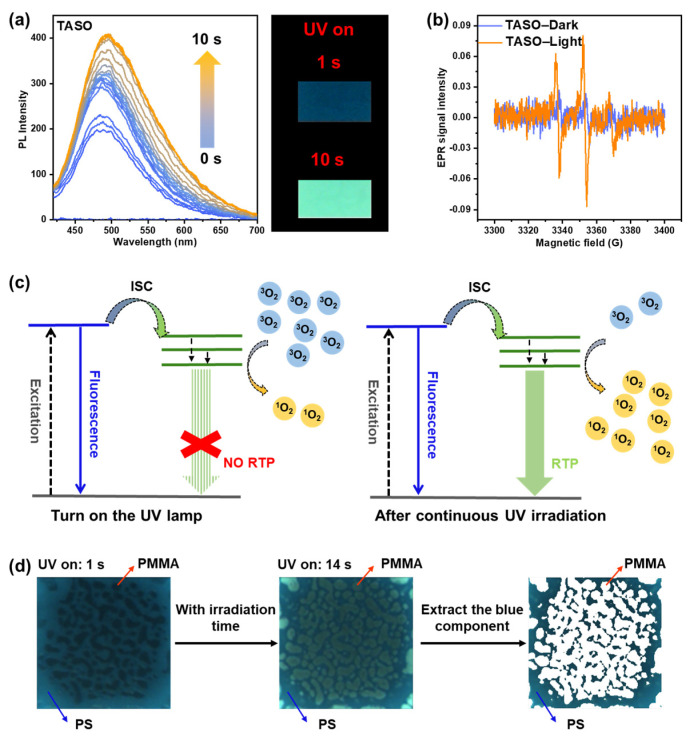
For the 1.0 wt.% TASO-doped PMMA film: (**a**) emission spectra and images under continuous 365 nm UV irradiation; (**b**) EPR spectra before (orange line) and after (blue line) UV irradiation was applied; (**c**) schematic diagram of UV-irradiation-induced RTP enhancement; (**d**) images of equal amounts of PS and PMMA blend polymer films (incorporated with 0.5 wt.% TASO) under continuous UV irradiation (365 nm, 8000 μW cm^−2^).

A fascinating phenomenon of UV-irradiation-induced RTP enhancement was observed in the TASO-doped PMMA films, and it was visible to the naked eye. When exposed to 365 nm UV irradiation, the doped PMMA film initially emitted relatively dim blue fluorescence. With prolonged irradiation, green phosphorescence gradually emerged, spreading throughout the film until the phosphorescence stabilized (Movie S1). This intriguing process can be monitored through continuous in situ emission spectra. As shown in Figure 4a, the emission intensity gradually increases with illumination time, reaching a peak at about 9 s. The maximum emission intensity after stabilization is 2.4 times higher than the initial intensity, confirming the excellent photo responsiveness of the TASO material induced by continuous UV irradiation. In addition, TA2O also exhibits photo responsiveness and displays a significant phosphorescence enhancement phenomenon, accompanied by an unchanged emission color (Figure S8a).

Since TASO and TA2O exhibit enhanced RTP under deoxygenated conditions, the observed UV-irradiation-induced RTP enhancement might be linked to oxygen consumption [41,42,43]. To validate this hypothesis, electron paramagnetic resonance (EPR) spectroscopy was employed (Figure 4b and Figure S8b). During the preparation of the PMMA-doped films, the singlet oxygen (^1^O_2_) scavenger, 2,2,6,6-tetramethylpiperidine (TEMP) [44], was incorporated. Following irradiation at 365 nm, a significant increase in the EPR signal intensity of TEMP-^1^O_2_ was observed, indicating efficient energy transfer from the photogenerated triplet excitons to oxygen molecules. Specifically, the generated triplet excitons sensitized the residual oxygen molecules (^3^O_2_) within the PMMA matrix, converting them into highly reactive singlet oxygen (^1^O_2_). The gradual consumption of oxygen molecules, combined with slower oxygen penetration into the PMMA matrix, creates a vacuum-like environment within the film, resulting in the dominance of RTP (Figure 4c). It is worth mentioning that TASO and TA2O have strong absorption bands at around 365 nm, enabling them to efficiently generate triplet excitons when exposed to commercial 365 nm UV lamp irradiation. Therefore, the observed UV-induced RTP enhancement was predominantly ascribed to the combined effect of effective UV absorption at specific wavelengths and the oxygen barrier properties of the polymer matrix, based on the efficient RTP of the materials. These characteristics highlight the promising potential applications of TASO and TA2O in various potential scenarios.

Utilizing the impressive UV-responsive dynamic RTP properties of TASO, we innovatively applied it to the detection of phase separation in polymer blends containing polymers with varying oxygen permeability. PMMA has strong oxygen barrier properties, which can ensure the UV-irradiation-induced RTP enhancement of TASO, while polystyrene (PS), with higher oxygen permeability, does not exhibit this characteristic (Movie S2). Thus, a new method for analyzing phase separation in binary blends of PMMA and PS can be developed using time-resolved, visualized optical imaging technology, which allows for the differentiation of PMMA and PS phases based on the distinct luminescence characteristics of the TASO contained in them.

To prepare a film for the phase separation detection experiment, we doped 0.5 wt.% TASO into equal amounts of blended (1:1 *w*/*w*) polymers of PS and PMMA. The blend was dissolved in chloroform, and a film was prepared on a silicon wafer via drop coating. Subsequently, the film was kept in a dark environment and allowed to dry at room temperature for 12 h prior to the phase separation detection experiment.

Following film preparation, the UV response process of the blend film, characterized ed by two distinct stages, was recorded with a camera (Figure 4d and Movie S3). In the first stage, under initial UV irradiation, the blend film exhibited inhomogeneous blue fluorescence. Notably, the emission intensity of the blue fluorescence varied across the film. We attribute the brighter blue regions to TASO residing within the PS phase (Figure S9), while the weaker blue regions likely correspond to TASO in the PMMA phase. This observed difference in emission intensity between PS and PMMA phases might be related to the varying rigidity of these two polymers. PS polymers are more rigid, so they can suppress non-radiative molecular motions more significantly, enabling the TASO molecules to show stronger emission. This stage can provide a ‘static’ state analysis of the phase separation in the blend film. In the second stage, as the irradiation continued, green phosphorescence emerged within the initially weak blue region, gradually intensifying. The brightness reached a peak after 12 s of irradiation and remained constant thereafter. The captured image (Figure 4d) reveals two distinct and clearly visible phase regions within the film, readily distinguishable even with the naked eye. This can be attributed to the fact that TASO in the PMMA region has the ability to undergo UV-irradiation-induced RTP enhancement due to the stronger oxygen barrier properties of the PMMA phase, while the TASO in in the PS region loses this ability due to the good oxygen permeability of the PS phase. This distinction in UV-irradiation-induced RTP enhancement between the TASO in the PMMA and PS phases further strengthens the analysis, i.e., a ‘dynamic’ state analysis, of the phase separation in the blend film. Furthermore, for a clearer and more intuitive visualization of the phase separation, we can analyze the video frames captured after the RTP emission stabilizes. By extracting the blue components from these frames, the distinction in emission color corresponding to TASO in the PS and PMMA phases is further accentuated. Overall, this method presents a novel “two-channel” (e.g., ‘static’ and ‘dynamic’) approach for analyzing phase separation in polymer blend films. It leverages a simple membrane production process (e.g., drop-coating) and requires only minimal quantities of RTP molecules. By combining these advantages with time-resolved detection, this method can achieve high accuracy in phase separation analysis.

## 3. Conclusions

In summary, utilizing a functional unit combination strategy, we designed and synthesized two new RTP molecules, TASO and TA2O, through incorporating a folded TA unit into the luminescent cores TA and AQ. The incorporation of TA significantly enhances the RTP performance of TASO and TA2O compared to their parent cores by effectively regulating the ISC process. When dispersed in a PMMA matrix and then exposed to continuous UV irradiation, TASO and TA2O exhibit dynamic RTP behavior. This phenomenon can be ascribed to the competitive relationship between the oxygen consumption rate within the PMMA matrix and the slower oxygen penetration rate, which facilitates the creation of a photo-induced oxygen-depleted microenvironment that results in the observed UV-irradiation-induced RTP enhancement. Leveraging the UV-radiation-induced RTP enhancement of TASO, we have creatively devised a novel time-resolved phase separation detection technology based on PS and PMMA blended polymers. This work not only provides a valuable strategy for designing photo-responsive RTP materials but also offers a feasible approach for visualizing phase separation in blend polymer films using time-resolved detection.

## Data Availability

The original contributions presented in the study are included in the article/Supplementary Materials; further inquiries can be directed to the corresponding authors.

## Supplementary Materials

The following supporting information can be downloaded at https://www.mdpi.com/article/10.3390/molecules29112621/s1. Scheme S1. Synthetic routes of TASO and TA2O. Figure S1. PMMA film doped with 1.0 wt.% AQ: (a) steady-state emission spectrum at room temperature and low temperature; (b) time-resolved emission spectrum at room temperature; (c) time-resolved emission spectrum at low temperature. Figure S2. Emission spectrum in diluted solvents with various polarity: (a) TA2O; (b) TASO. Figure S3. Temperature-dependent test of 1.0 wt.% TASO doped PMMA film: (a) steady-state emission spectrum; (b) time-resolved emission spectrum at 440 nm; (c) time-resolved emission spectrum at 523 nm. Figure S4. Diluted THF solution (10^−5^ mol L^−1^) of TASO at low temperature: (a) steady-state emission spectrum; (b) time-resolved emission spectrum at 515 nm; (c) time-resolved emission spectrum at 450 nm. Figure S5. Temperature-changing test of PMMA film doped with 1.0 wt.% TA2O: (a) steady-state emission spectrum; (b) time-resolved emission spectrum. Figure S6. Diluted THF solution (10^−5^ mol L^−1^) of TA2O at low-temperature: (a) steady-state emission spectrum; (b) time-resolved emission spectrum. Figure S7. SOC and energy level distribution: (a) TX; (b) AQ. Figure S8. 1.0 wt.% TA2O doped PMMA film: (a) emission spectrum under continuous 365 nm UV irradiation; (b) EPR spectra before (orange line) and after (blue line) UV irradiation. Figure S9. 1.0 wt.% TASO doped PS film: (a) emission spectrum before and after deoxygenation; (b) and (c) time-resolved emission spectra before deoxygenation; (c) time-resolved emission spectrum after deoxygenation. Movie S1. Luminescent behavior of TASO-doped PMMA films under continuous UV irradiation. Movie S2. Luminescent behavior of TASO-doped PS films under continuous UV irradiation. Movie S3. Luminescent behavior of TASO-doped PMMA and PS blend films under continuous UV irradiation. References [45,46,47,48,49,50,51,52,53,54] are cited in the Supplementary Materials.

## References

[1] Lee D.R., Lee K.H., Shao W., Kim C.L., Kim J., Lee J.Y. (2020). Heavy Atom Effect of Selenium for Metal-Free Phosphorescent Light-Emitting Diodes. Chem. Mater..

[2] Chen Z.J., Li M.K., Gu Q., Peng X.M., Qiu W.D., Xie W.T., Liu D.H., Jiao Y.H., Liu K.K., Zhou J.D. (2023). Highly Efficient Purely Organic Phosphorescence Light-Emitting Diodes Employing a Donor-Acceptor Skeleton with a Phenoxaselenine Donor. Adv. Sci..

[3] Gu L., Wu H.W., Ma H.L., Ye W.P., Jia W.Y., Wang H., Chen H.Z., Zhang N., Wang D.D., Qian C. (2020). Color-tunable ultralong organic room temperature phosphorescence from a multicomponent copolymer. Nat. Commun..

[4] Deng Y.C., Li P., Sun S.J., Jiang H.Y., Ji X., Li H.R. (2020). Proton-Activated Amorphous Room-Temperature Phosphorescence for Humidity Sensing and High-Level Data Encryption. Chem. Asian J..

[5] Feng Y., Cheng J.H., Zhou L., Zhou X.G., Xiang H.F. (2012). Ratiometric optical oxygen sensing: A review in respect of material design. Analyst.

[6] Skuodis E., Leitonas K., Panchenko A., Volyniuk L., Keruckiene R., Volyniuk D., Minaev B.F., Grazulevicius J.V. (2022). Very sensitive probes for quantitative and organoleptic detection of oxygen based on conformer-induced room-temperature phosphorescence enhancement of the derivative of triazatruxene and phenothiazine. Sens. Actuat. B-Chem..

[7] Hou Y.Z., Jiang G.Y., Gong J.Y., Sha R., Wang J.G. (2021). Recent Advances of Pure Organic Room Temperature Phosphorescence Materials for Bioimaging Applications. Chem. Res. Chin. Univ..

[8] Fan Y.Y., Liu S.W., Wu M., Xiao L.Y., Fan Y.H., Han M.M., Chang K., Zhang Y.F., Zhen X., Li Q.Q. (2022). Mobile Phone Flashlight-Excited Red Afterglow Bioimaging. Adv. Mater..

[9] Zhen X., Tao Y., An Z.F., Chen P., Xu C.J., Chen R.F., Huang W., Pu K.Y. (2017). Ultralong Phosphorescence of Water-Soluble Organic Nanoparticles for In Vivo Afterglow Imaging. Adv. Mater..

[10] Zhou W.L., Lin W.J., Chen Y., Liu Y. (2022). Supramolecular assembly confined purely organic room temperature phosphorescence and its biological imaging. Chem. Sci..

[11] Ma X., Wang J., Tian H. (2019). Assembling-Induced Emission: An Efficient Approach for Amorphous Metal-Free Organic Emitting Materials with Room Temperature Phosphorescence. Acc. Chem. Res..

[12] Yan X., Peng H., Xiang Y., Wang J., Yu L., Tao Y., Li H.H., Huang W., Chen R.F. (2022). Recent Advances on Host-Guest Material Systems toward Organic Room Temperature Phosphorescence. Small.

[13] Ward J.S., Nobuyasu R.S., Batsanov A.S., Data P., Monkman A.P., Dias F.B., Bryce M.R. (2016). The interplay of thermally activated delayed fluorescence (TADF) and room temperature organic phosphorescence in sterically-constrained donor-acceptor charge-transfer molecules. Chem. Commun..

[14] El-Sayed M.A. (1963). Comments on Contaminating the Ground State with Triplet Character. J. Chem. Phys..

[15] Kuila S., George S.J. (2020). Phosphorescence Energy Transfer: Ambient Afterglow Fluorescence from Water-Processable and Purely Organic Dyes via Delayed Sensitization. Angew. Chem. Int. Edit..

[16] Wang S., Cheng Z.Q., Han X.C., Shu H.Y., Wu X.F., Tong H., Wang L.X. (2022). Efficient and tunable purely organic room temperature phosphorescence films from selenium-containing emitters achieved by structural isomerism. J. Mater. Chem. C..

[17] Ma X., Xu C., Wang J., Tian H. (2018). Amorphous Pure Organic Polymers for Heavy-Atom-Free Efficient Room-Temperature Phosphorescence Emission. Angew. Chem. Int. Edit..

[18] Zhou Z.X., Mao Z., Yang Z., Yang T.T., Zhu L.J., Long Y.B., Chi Z.G., Liu S.W., Aldred M.P., Chen X.D. (2021). Achieving white-light emission in a single-component polymer with halogen-assisted interaction. Sci. China Chem..

[19] Ma L.W., Sun S.Y., Ding B.B., Ma X., Tian H. (2021). Highly Efficient Room-Temperature Phosphorescence Based on Single-Benzene Structure Molecules and Photoactivated Luminescence with Afterglow. Adv. Funct. Mater..

[20] Xie Z.L., Zhang X.Y., Wang H.L., Huang C., Sun H.D., Dong M.Y., Ji L., An Z.F., Yu T., Huang W. (2021). Wide-range lifetime-tunable and responsive ultralong organic phosphorescent multi-host/guest system. Nat. Commun..

[21] Gu L., Shi H.F., Gu M.X., Ling K., Ma H.L., Cai S.Z., Song L.L., Ma C.Q., Li H., Xing G.C. (2018). Dynamic Ultralong Organic Phosphorescence by Photoactivation. Angew. Chem. Int. Edit..

[22] Wang Y.S., Yang J., Fang M.M., Gong Y.X., Ren J., Tu L.J., Tang B.Z., Li Z. (2021). New Phenothiazine Derivatives That Exhibit Photoinduced Room-Temperature Phosphorescence. Adv. Funct. Mater..

[23] Huang Q.Q., Mei X.F., Xie Z.L., Wu D.B., Yang S.M., Gong W.J., Chi Z.G., Lin Z.H., Ling Q.D. (2019). Photo-induced phosphorescence and mechanoluminescence switching in a simple purely organic molecule. J. Mater. Chem. C..

[24] Yang Z.Q., Liu H.C., Zhang X.Y., Lv Y.B., Fu Z.Y., Zhao S.Q., Liu M., Zhang S.T., Yang B. (2023). Photo-Responsive Dynamic Organic Room-Temperature Phosphorescence Materials Based on a Functional Unit Combination Strategy. Adv. Mater..

[25] Thomas H., Pastoetter D.L., Gmelch M., Achenbach T., Schlögl A., Louis M., Feng X.L., Reineke S. (2020). Aromatic Phosphonates: A Novel Group of Emitters Showing Blue Ultralong Room Temperature Phosphorescence. Adv. Mater..

[26] Li Q.Q., Li Z. (2020). Molecular Packing: Another Key Point for the Performance o Organic and Polymeric Optoelectronic Materials. Acc. Chem. Res..

[27] Liu X.W., Zhao W.J., Wu Y., Meng Z.O., He Z.K., Qi X., Ren Y.R., Yu Z.Q., Tang B.Z. (2022). Photo-thermo-induced room-temperature phosphorescence through solid-state molecular motion. Nat. Commun..

[28] Li Y.H., Gu F., Ding B.B., Zou L., Ma X. (2021). Photo-controllable room-temperature phosphorescence of organic photochromic polymers based on hexaarylbiimidazole. Sci. China Chem..

[29] Su Y., Phua S.Z.F., Li Y.B., Zhou X.J., Jana D., Liu G.F., Lim W.Q., Ong W.K., Yang C.L., Zhao Y.L. (2018). Ultralong room temperature phosphorescence from amorphous organic materials toward confidential information encryption and decryption. Sci. Adv..

[30] Jia X.Y., Shao C.C., Bai X., Zhou Q.H., Wu B., Wang L.J., Yue B.B., Zhu H.M., Zhu L.L. (2019). Photoexcitation-controlled self-recoverable molecular aggregation for flicker phosphorescence. Proc. Nat. Acad. Sci. USA.

[31] Jia X.Y., Zhu L.L. (2023). Photoexcitation-Induced Assembly: A Bottom-Up Physical Strategy for Driving Molecular Motion and Phase Evolution. Acc. Chem. Res..

[32] Zhao S.Q., Yang Z.Q., Zhang X.Y., Liu H.C., Lv Y.B., Wang S.Y., Yang Z.Z., Zhang S.T., Yang B. (2023). A functional unit combination strategy for enhancing red room-temperature phosphorescence. Chem. Sci..

[33] Liu H.C., Gao Y., Cao J.G., Li T.X., Wen Y.T., Ge Y.P., Zhang L.L., Pan G.C., Zhou T., Yang B. (2018). Efficient room-temperature phosphorescence based on a pure organic sulfur-containing heterocycle: Folding-induced spin-orbit coupling enhancement. Mater. Chem. Front..

[34] Qiu W.D., Cai X.Y., Chen Z.J., Wei X.F., Li M.K., Gu Q., Peng X.M., Xie W.T., Jiao Y.H., Gan Y.Y. (2022). A “Flexible” Purely Organic Molecule Exhibiting Strong Spin–Orbital Coupling: Toward Nondoped Room-Temperature Phosphorescence OLEDs. J. Phys. Chem. Lett..

[35] Li M.K., Xie W.T., Cai X.Y., Peng X.M., Liu K.K., Gu Q., Zhou J.D., Qiu W.D., Chen Z.J., Gan Y.Y. (2022). Molecular Engineering of Sulfur-Bridged Polycyclic Emitters Towards Tunable TADF and RTP Electroluminescence. Angew. Chem. Int. Ed..

[36] Liu H.C., Pan G.C., Yang Z.Q., Wen Y.T., Zhang X.Y., Zhang S.T., Li W.J., Yang B. (2022). Dual-Emission of Fluorescence and Room-Temperature Phosphorescence for Ratiometric and Colorimetric Oxygen Sensing and Detection Based on Dispersion of Pure Organic Thianthrene Dimer in Polymer Host. Adv. Opt. Mater..

[37] Wen Y.T., Liu H.C., Zhang S.T., Gao Y., Yan Y., Yang B. (2019). One-dimensional π-π stacking induces highly efficient pure organic room-temperature phosphorescence and ternary-emission single-molecule white light. J. Mater. Chem. C..

[38] Ong W.J., Swager T.M. (2018). Dynamic self-correcting nucleophilic aromatic substitution. Nat. Chem..

[39] Grabowski Z.R., Rotkiewicz K. (2003). Structural Changes Accompanying Intramolecular Electron Transfer: Focus on Twisted Intramolecular Charge-Transfer States and Structures. Chem. Rev..

[40] Uoyama H., Goushi K., Shizu K., Nomura H., Adachi C. (2012). Highly efficient organic light-emitting diodes from delayed fluorescence. Nature.

[41] Zang L., Shao W., Kwon M.S., Zhang Z., Kim J. (2020). Photoresponsive Luminescence Switching of Metal-Free Organic Phosphors Doped Polymer Matrices. Adv. Opt. Mater..

[42] Gmelch M., Thomas H., Fries F., Reineke S. (2019). Programmable transparent organic luminescent tags. Sci. Adv..

[43] Louis M., Thomas H., Gmelch M., Haft A., Fries F., Reineke S. (2019). Blue-Light-Absorbing Thin Films Showing Ultralong Room-Temperature Phosphorescence. Adv. Mater..

[44] Wang S., Sun Q., Chen W., Tang Y.Q., Aguila B., Pan Y.X., Zheng A.M., Yang Z.Y., Wojtas L., Ma S.Q. (2020). Programming Covalent Organic Frameworks for Photocatalysis: Investigation of Chemical and Structural Variations. Matter.

[45] Frisch M.J., Trucks G.W., Schlegel H.B., Scuseria G.E., Robb M.A., Cheeseman J.R., Scalmani G., Barone V., Petersson G.A., Nakatsuji H. (2016). Gaussian 16, Revision B.01.

[46] Liu W.J., Hong G.Y., Dai D.D., Li L.M., Dolg M. (1997). The Beijing four-component density functional program package (BDF) and its application to EuO, EuS, YbO and YbS. Theor. Chem. Acc..

[47] Zhang Y., Suo B.B., Wang Z.K., Zhang N., Li Z.D., Lei Y.B., Zou W.L., Gao J., Peng D.L., Pu Z.C. (2020). BDF: A relativistic electronic structure program package. J. Chem. Phys..

[48] Liu W.J., Wang F., Li L.M. (2003). The Beijing Density Functional (BDF) Program Package: Methodologies and Applications. J. Theor. Comput. Chem..

[49] Liu W.J., Wang F., Li L.M. (2004). Recent Advances in Relativistic Molecular Theory. Encyclopedia of Computational Chemistry.

[50] Li Z.D., Suo B.B., Zhang Y., Xiao Y.L., Liu W.J. (2013). Combining spin-adapted open-shell TD-DFT with spin–orbit coupling. Mol. Phys..

[51] Li Z.D., Xiao Y.L., Liu W.J. (2012). On the spin separation of algebraic two-component relativistic Hamiltonians. J. Chem. Phys..

[52] Li Z.D., Xiao Y.L., Liu W.J. (2014). On the spin separation of algebraic two-component relativistic Hamiltonians: Molecular properties. J. Chem. Phys..

[53] Hayashi H., Aratani N., Yamada H. (2017). Semiconducting Self-Assembled Nanofibers Prepared from Photostable Octafluorinated Bisanthene Derivatives. Chem.-Eur. J..

[54] Guan X.Y., Li H., Ma Y.C., Xue M., Fang Q.R., Yan Y.S., Valtchev V., Qiu S.L. (2019). Chemically stable polyarylether-based covalent organic frameworks. Nat. Chem..

