# Supplementary material for: Combining Functional Units to Design Organic Materials with Dynamic Room-Temperature Phosphorescence under Continuous Ultraviolet Irradiation
Source: Molecules. 2024 Jun 2;29(11):2621. doi: 10.3390/molecules29112621 (PMC11173552; doi:10.3390/molecules29112621)
Supplement: Supplementary file 1 [file molecules-29-02621-s001.zip › supporting_information.pdf]

Supporting Information

Combining Functional Units to Design Organic Materials with Dynamic Room-Temperature Phosphorescence under Continuous Ultraviolet Irradiation

Meng Liu [1,†], Zhiqiang Yang [1,†], Zhe Feng [1,†], Ningyuan Zhao [2], Ruihua Bian [2], Jinpu Wu [2], Qing Yang [3], Shuaiqiang Zhao [1], Haichao Liu [1,*] and Bing Yang [1,*]

[1] State Key Laboratory of Supramolecular Structure and Materials, College of Chemistry, Jilin Univer-sity, Changchun 130012, P.R. China.

[2] College of Chemistry, Jilin University, Changchun 130012, P.R. China.

[3] State Key Laboratory of Superhard Materials, College of Physics, Jilin University, Changchun 130012, P.R. China.

* Correspondence: hcliu@jlu.edu.cn, yangbing@jlu.edu.cn.

† These authors contributed equally to this work.

SI-Experimental Procedures

Structural characterization

^{1}H NMR and ^{13}C NMR spectra were obtained from a Bruker AVANCE 500 spectrometer with tetramethylsilane (TMS) as the internal standard. Mass spectra were acquired using a Thermo Fisher ITQ1100 instrument.

Photophysical measurements

The ultraviolet-visible (UV-vis) absorption spectra of the solutions was measured with a PerkinElmer LAMBDA 365 spectrophotometer. The emission and time-resolved emission spectra were recorded using a FLS980 fluorescence spectrometer. The emission spectra under continuous 365 nm UV irradiation were collected using an Ocean Optics QE series spectrometer. Electron paramagnetic resonance (EPR) spectra were collected using a Bruker E500 EPR spectrometer. The photoluminescence quantum yields (PLQYs) were determined using an integrating sphere on the FLS980 fluorescence spectrometer. The PLQYs in vacuum were calculated by comparing the spectral integration areas in vacuum and air. Temperature-dependent emission and time-resolved emission spectra were acquired in vacuum using a FLSP980 fluorescence spectrometer equipped with a chamber (Optistat DN2, OXFORD INSTRUMENTS) and a vacuum pump. Liquid nitrogen and vacuum pump were employed for low-temperature or temperature-dependent experiments, in conjunction with the instrument's temperature control program for precise temperature adjustments.

Theoretical calculations

Gaussian 16 (version A.03) software package[1] was used on a PowerLeader cluster for molecular geometry optimization, energy level analysis, and natural transition orbitals (NTOs) analysis. Ground-state geometries was optimized using B3LYP/6-31G(d, p), followed by simulation and evaluation of NTOs to identify the contribution of the dominant 'hole'–'particle' pair. Spin–orbit coupling (SOC) coefficients were calculated using Beijing Density Function (BDF) software[2] based on TD-B3LYP/6-31G(d, p).

SII-Synthesis Details

The synthetic routes of the target compounds are presented as follows (Scheme S1).

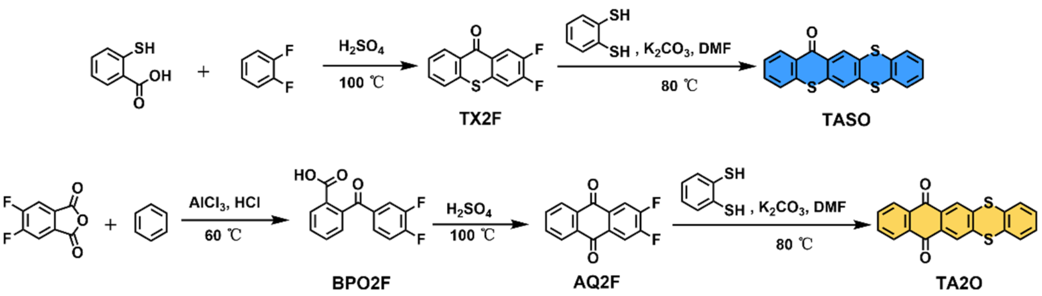

Scheme S1. Synthetic routes of TASO and TA2O.

Synthesis of 14H-thiochromeno[2,3-b]thianthren-14-one (TASO)

α-Mercaptobenzoic acid (1.00 g, 6.50 mmol) and 1,2-difluorobenzene (0.50 ml, 4.80 mmol) were dissolved in concentrated sulfuric acid (8 ml, 98%), followed by stirring at room temperature for 24 h. The mixture was then heated at 100 °C for 1 h, during which the color of the mixture changed from yellow to dark red. Subsequently, the mixture was cooled to room temperature and poured into ice water. The resulting precipitate was collected by vacuum filtration and washed. The crude product was solved and extracted with chloroform, the organic extract was dried over anhydrous magnesium sulfate, and the solvent was removed. Isolation of the product was achieved through silica gel column chromatography using dichloromethane/petroleum ether. Finally, a white solid, TX2F, was obtained (0.82 g, 51% yield). ^{1}H NMR (500 MHz, DMSO-d_6, 25 °C, TMS): δ 8.48 (dd, J = 8.1, 1.6 Hz, 1H), 8.38 (dd, J = 11.3, 8.4 Hz, 1H), 8.21 (dd, J = 10.9, 7.2 Hz, 1H), 7.91 (d, J = 8.1 Hz, 1H), 7.86 – 7.79 (m, 1H), 7.64 (t, J = 7.6 Hz, 1H). GC-MS, EI, mass m/z: 248.13 [M^+].

Under nitrogen conditions, 1,2-benzenedithiol (0.27 g, 2.00 mmol), TX2F (0.50 g, 2.00 mmol), and K_2CO_3 (0.83 g, 6.00 mmol) were added to 30 ml of *N*, *N*-dimethyl formamide (DMF). The mixture was stirred and heated to 80 °C overnight, then cooled to room temperature. After removing the solvent by distillation under reduced pressure, silica gel chromatography was performed using a mixed solvent of petroleum ether/dichloromethane as eluent. Finally, a yellow solid, TASO (0.46 g, 65% yield), was obtained. ^{1}H NMR (500 MHz, CD_2Cl_2, 25 °C, TMS): δ 8.67 (s, 1H), 8.59 (dd, J = 8.2, 1.5 Hz, 1H), 7.77 (s, 1H), 7.73 – 7.62 (m, 2H), 7.62 – 7.51 (m, 3H), 7.39 – 7.31 (m, 2H). ^{13}C NMR (126 MHz, $CDCl_3$, 25 °C, TMS): δ 178.94, 141.78, 136.72, 136.52, 134.90, 133.95, 133.60, 132.54, 129.98, 129.06, 128.84, 128.30, 128.08, 126.60, 126.09, 124.96. GC-MS, EI, mass m/z: 346.82 [M^+].

Synthesis of naphtho[2,3-b]thianthrene-7,12-dione (TA2O)

AQ2F was synthesized according to literature reports[3]. ^{1}H NMR (500 MHz, $CDCl_3$, 25 °C, TMS): δ 8.34 (dd, J = 5.8, 3.3 Hz, 2H), 8.13 (t, J = 8.7 Hz, 2H), 7.86 (dd, J = 5.8, 3.3 Hz, 2H). GC-MS, EI, mass m/z: 245.01 [M^+].

The synthesis methods of TASO and TA2O are similar. The final orange solid TA2O (0.75 g, yield 72%) was obtained. ^{1}H NMR (500 MHz, CD_2Cl_2, 25 °C, TMS): δ 8.36 (s, 2H), 8.32 (dd, J = 5.8, 3.3 Hz, 2H), 7.87 (dd, J = 5.8, 3.3 Hz, 2H), 7.56 (dd, J = 5.8, 3.3 Hz, 2H), 7.37 (dd, J = 5.8, 3.3 Hz, 2H). ^{13}C NMR (126 MHz, $CDCl_3$, 25 °C, TMS): δ 182.18, 143.02, 134.33, 134.27, 133.39, 132.36, 128.84, 128.51, 127.35, 126.83. GC-MS, EI, mass m/z: 350.71[M^+].

SIII-Figures

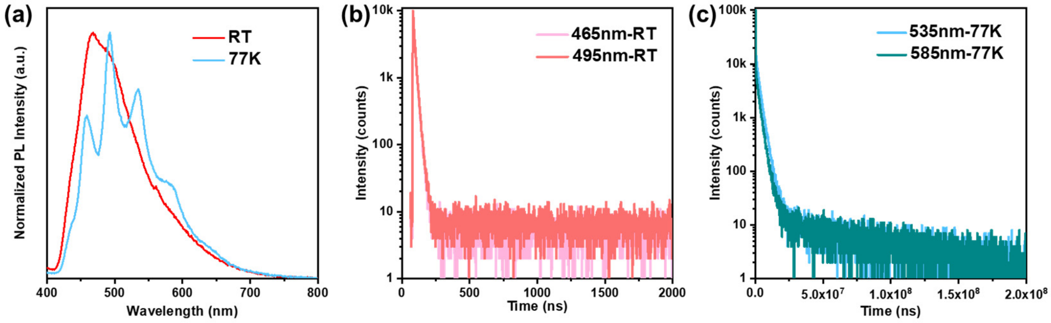

Figure S1. PMMA film doped with 1.0 wt% AQ: (a) steady-state emission spectrum at room temperature and low temperature; (b) time-resolved emission spectrum at room temperature; (c) time-resolved emission spectrum at low temperature.

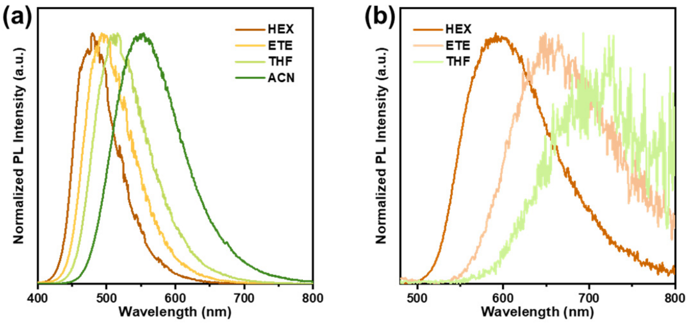

Figure S2. Emission spectrum in diluted solvents with various polarity: (a) TA2O; (b) TASO. HEX is hexane, ETE is ethyl ether, THF is tetrahydrofuran, and ACN is acetonitrile.

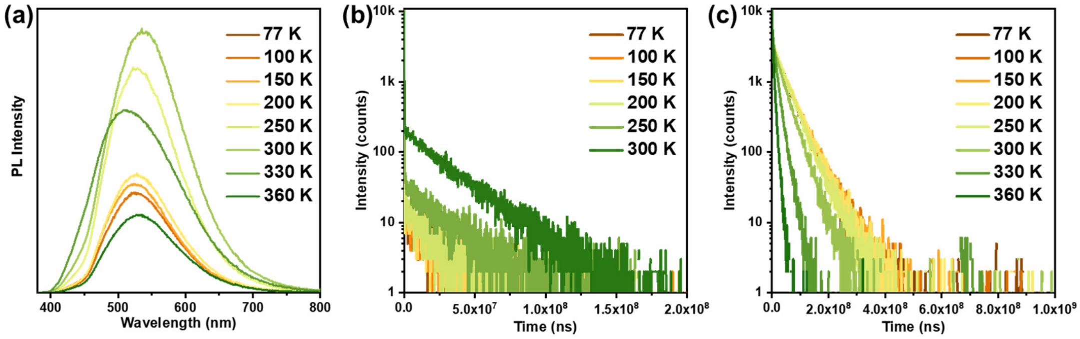

Figure S3. Temperature-dependent test of 1.0 wt.% TASO doped PMMA film: (a) steady-state emission spectrum; (b) time-resolved emission spectrum at 440 nm; (c) time-resolved emission spectrum at 523 nm.

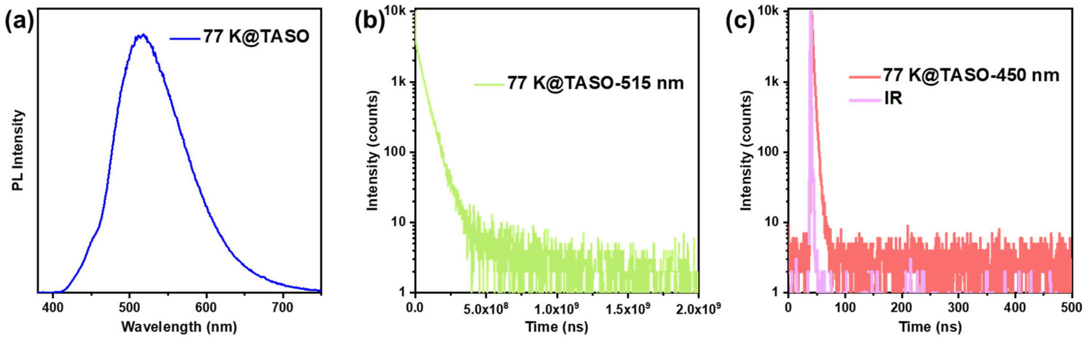

Figure S4. Diluted THF solution (10^{-5} mol L^{-1}) of TASO at low temperature: (a) steady-state emission spectrum; (b) time-resolved emission spectrum at 515 nm; (c) time-resolved emission spectrum at 450 nm.

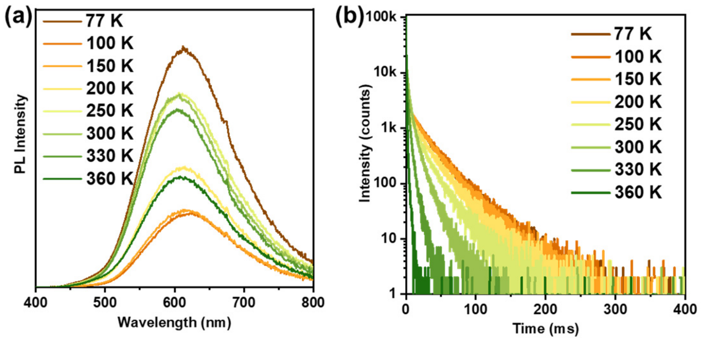

Figure S5. Temperature-changing test of PMMA film doped with 1.0 wt% TA2O: (a) steady-state emission spectrum; (b) time-resolved emission spectrum.

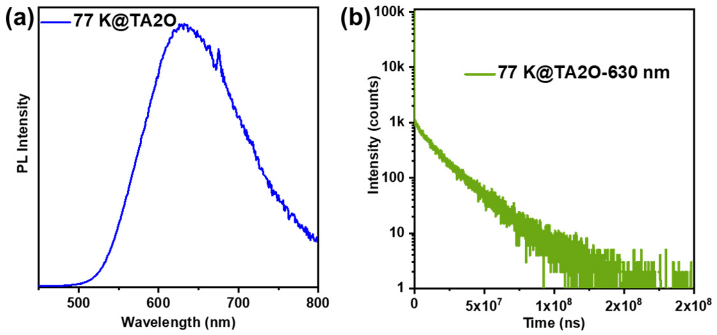

Figure S6. Diluted THF solution (10^{-5} mol L^{-1}) of TA2O at low-temperature: (a) steady-state emission spectrum; (b) time-resolved emission spectrum.

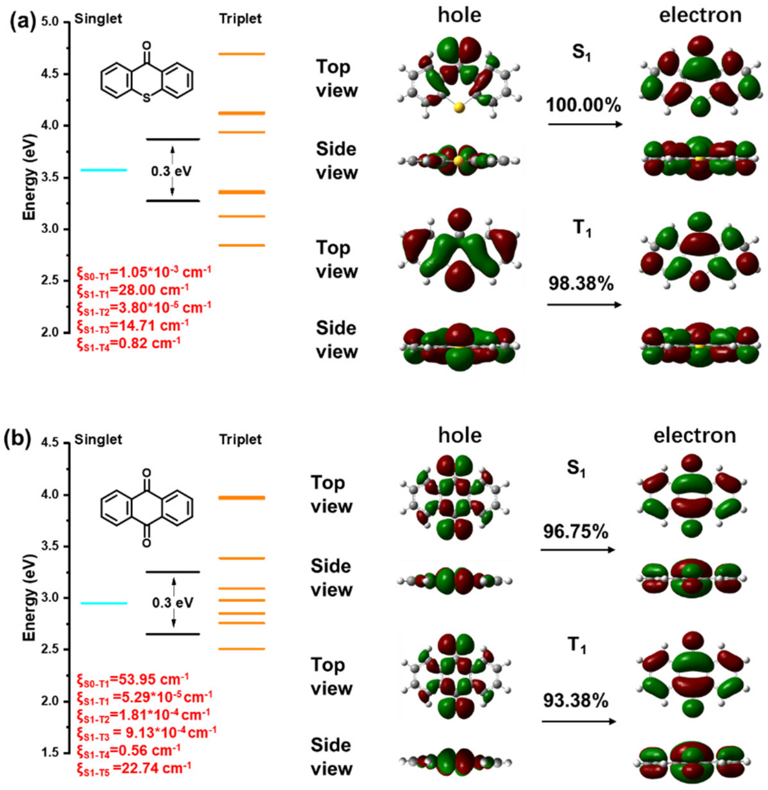

Figure S7. SOC and energy level distribution: (a) TX; (b) AQ.

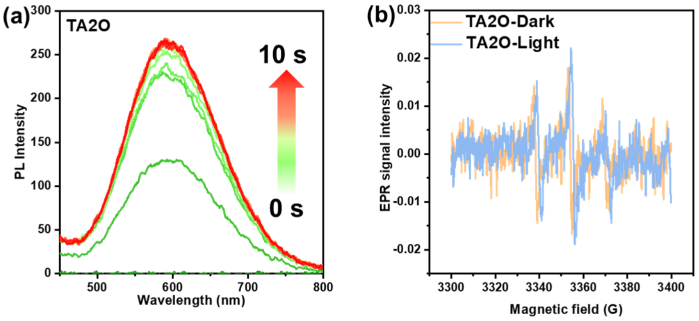

Figure S8. 1.0 wt.% TA2O doped PMMA film: (a) emission spectrum under continuous 365 nm UV irradiation; (b) EPR spectra before (orange line) and after (blue line) UV irradiation.

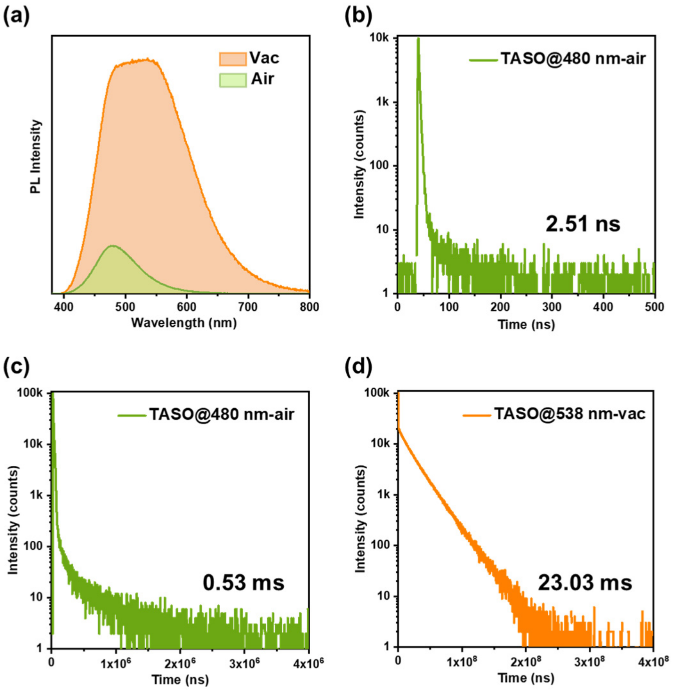

Figure S9. 1.0 wt.% TASO doped PS film: (a) emission spectrum before and after deoxygenation; (b) and (c) time-resolved emission spectra before deoxygenation; (c) time-resolved emission spectrum after deoxygenation.

SIV-References

[1] M. J. Frisch, G. W. Trucks, H. B. Schlegel, G. E. Scuseria, M. A. Robb, J. R. Cheeseman, G. Scalmani, V. Barone, G. A. Petersson, H. Nakatsuji, X. Li, M. Caricato, A. V. Marenich, J. Bloino, B. G. Janesko, R. Gomperts, B. Mennucci, H. P. Hratchian, J. V. Ortiz, A. F. Izmaylov, J. L. Sonnenberg, Williams, F. Ding, F. Lipparini, F. Egidi, J. Goings, B. Peng, A. Petrone, T. Henderson, D. Ranasinghe, V. G. Zakrzewski, J. Gao, N. Rega, G. Zheng, W. Liang, M. Hada, M. Ehara, K. Toyota, R. Fukuda, J. Hasegawa, M. Ishida, T. Nakajima, Y. Honda, O. Kitao, H. Nakai, T. Vreven, K. Throssell, J. A. Montgomery Jr., J. E. Peralta, F. Ogliaro, M. J. Bearpark, J. J. Heyd, E. N. Brothers, K. N. Kudin, V. N. Staroverov, T. A. Keith, R. Kobayashi, J. Normand, K. Raghavachari, A. P. Rendell, J. C. Burant, S. S. Iyengar, J. Tomasi, M. Cossi, J. M. Millam, M. Klene, C. Adamo, R. Cammi, J. W. Ochterski, R. L. Martin, K. Morokuma, O. Farkas, J. B. Foresman, D. J. Fox, Wallingford, CT, **2016**.

[2] (a) W. Liu, G. Hong, D. Dai, L. Li, M. Dolg, *Theor. Chem. Acc.* **1997**, *96*, 75-83; (b) Y. Zhang, B. Suo, Z. Wang, N. Zhang, Z. Li, Y. Lei, W. Zou, J. Gao, D. Peng, Z. Pu, Y. Xiao, Q. Sun, F. Wang, Y. Ma, X. Wang, Y. Guo, W. Liu, *J. Chem. Phys.* **2020**, *152*, 064113; (c) W. Liu, F. Wang, L. Li, *J. Theor. Comput. Chem.* **2003**, *02*, 257-272; (d) W. Liu, F. Wang, L. Li, in *Recent Advances in Relativistic Molecular Theory*, pp. 257-282; (e) Z. Li, B. Suo, Y. Zhang, Y. Xiao, W. Liu, *Mol. Phys.* **2013**, *111*, 3741-3755; (f) Z. Li, Y. Xiao, W. Liu, *J. Chem. Phys.* **2012**, *137*,154114; (g) Z. Li, Y. Xiao, W. Liu, *J. Chem. Phys.* **2014**, *141*, 054111.

[3] (a) H. Hayashi, N. Aratani, H. Yamada, *Chem.-Eur. J.* **2017**, *23*, 7000-7008; (b) X. Guan, H. Li, Y. Ma, M. Xue, Q. Fang, Y. Yan, V. Valtchev, S. Qiu, *Nat. Chem.* **2019**, *11*, 587-594.
